# Carotid Intima‐Media Thickness, Carotid Distensibility, and Incident Heart Failure in Older Men: The British Regional Heart Study

**DOI:** 10.1161/JAHA.124.037167

**Published:** 2025-03-21

**Authors:** Atinuke Akinmolayan, A. Olia Papacosta, Lucy T. Lennon, Elizabeth A. Ellins, Julian P. J. Halcox, Peter H Whincup, S. Goya Wannamethee

**Affiliations:** ^1^ Department of Primary Care and Population Health University College London London United Kingdom; ^2^ Institute of Life Science, Swansea University Swansea United Kingdom; ^3^ Population Health Research Institute, St George’s University of London London United Kingdom

**Keywords:** cardiovascular diseases, carotid artery distensibility, carotid intima‐media thickness, heart failure, Heart Failure, Cardiovascular Disease, Epidemiology, Prognosis

## Abstract

**Background:**

Carotid intima‐media thickness (CIMT) and carotid distensibility are markers of arterial change; however, little is known of the association with incident heart failure (HF). We aimed to assess this.

**Methods:**

This was a longitudinal analysis of data from the British Regional Heart Study, a prospective cohort study. A total of 1631 men aged 71 to 92 years, without a diagnosis of HF at baseline, were included. Between 2010 and 2012, participants completed a questionnaire, underwent a physical examination, and provided a fasting blood sample. CIMT and carotid artery distension were measured, and carotid distensibility was calculated. Cox proportional hazards modeling was used to assess the multivariate‐adjusted hazard ratios (HRs) of incident HF by quartiles of CIMT and distensibility, excluding men with prevalent myocardial infarction.

**Results:**

The values used in the analysis were adjusted for age, social class, smoking, physical activity, alcohol status, body mass index, use of statins and antihypertensives, prevalent diabetes and stroke, pulse pressure, and presence of atrial arrhythmias. Lower carotid distensibility (bottom quartile) and higher CIMT (top quartile) were associated with increased risk of incident HF (HR, 2.55 [95% CI, 1.24–5.24]; *P*=0.01; and HR, 2.20 [95% CI, 1.14–4.23]; *P*=0.02, respectively). CIMT but not carotid distensibility was associated with incident myocardial infarction. The association between carotid distensibility and incident HF persisted after adjustment for incident myocardial infarction and CIMT (HR, 2.53 [95% CI, 1.23–5.22]; *P*=0.01); however, the association between CIMT and incident HF was attenuated after this adjustment (HR, 1.64 [95% CI, 0.84–3.21]; *P*=0.15).

**Conclusions:**

Lower carotid distensibility and higher CIMT were associated with an increased risk of incident HF, despite adjustment for incident myocardial infarction.

**Nonstandard Abbreviations and Acronyms**
CIMT: carotid intima‐media thickness
CV: coefficient of variation

**Research Perspective**

**What Is New?**

- There have been conflicting reports about the association between carotid intima‐media thickness (CIMT), carotid distensibility, and incident heart failure (HF); CIMT has been shown to be associated with incident HF, but there remains uncertainty about carotid distensibility.
- In our population of British men, aged 71 to 92 years, increased CIMT and lower carotid distensibility are associated with incident HF, after adjustment for traditional cardiovascular risk factors, CIMT, and incident myocardial infarction; CIMT but not carotid distensibility is associated with incident myocardial infarction.

**What Question Should Be Addressed Next?**

- What measures of CIMT (mean CIMT or maximum CIMT) are better associated with incident myocardial infarction in the older adult population?
- Does the association between carotid distensibility and HF vary according to HF subtype in the older adult population?

In the United Kingdom, heart failure (HF) affects ≈920 000 people, with 200 000 new cases annually.^1^ Primarily a disease affecting older adults, at least 5% of people aged >75 years are affected with HF, with prevalence increasing with age to 15% in people aged >85 years.^2^ It is the only cardiovascular disease (CVD) increasing in prevalence despite advances in medical care, likely due to HF being a final complication for several cardiac conditions that affect cardiac contractility.^3^ Given the aging of many developed‐country populations and increased survival from acute cardiac events, those affected by HF are often debilitated by multimorbidity, which in turn contributes to an increased mortality rate, use of public health resources, and health care cost.^3^ Despite advances in medical care, HF remains a progressive and incurable chronic disease with significant impacts on the individual, their family, and society. Thus, effective preventative strategies are urgently needed.

Although the typical onset of HF appears later in life, evidence suggests that subclinical vascular modifications develop much earlier. Early, accurate diagnosis is critical to improving HF outcomes. On a structural level, HF is characterized by a combination of reduced contractility, ventricular remodeling, systolic or diastolic dysfunction, ultimately leading to the inability of the heart to meet the circulatory demands of the body. Atherosclerosis is a common cause of HF,^4^ affecting the coronary as well as the carotid arteries. Development of atherosclerosis is a gradual process with a long subclinical state before it clinically manifests as CVD. Research into arterial mechanical properties have led to new parameters for predicting cardiovascular events.^5^, ^6^ Increased arterial pulse wave velocity, a marker of arterial stiffness, has been shown to be a strong predictor of CVD events,^7^ but reports have shown it not to be independently associated with risk of HF after adjustment for traditional CVD risk factors in older adults.^8^ Given the structural and functional heterogeneity of arteries, especially with age,^9^ many studies focus on the aorta and common carotid artery. The common carotid artery can be examined noninvasively with ultrasound, with a high degree of reproducibility^10^; however, there are few prospective studies that explore the relationship between carotid wall hemodynamics and HF events.

Increased carotid intima‐media thickness (CIMT) is a feature of arterial wall aging and is reported as being a marker of arterial injury.^11^ It has been shown to be associated with incident HF, possibly due to shared atherosclerotic pathways or through its relationship with arterial distensibility.^12^, ^13^, ^14^ Arterial distensibility is a measure of the arterial ability to expand and contract in response to cardiac pulsation and relaxation.^15^ Reduced arterial (carotid and aortic) distensibility, indicators of vascular structure and function, have been shown to be early markers of subclinical vascular modification; however, little is known of the association with incident CVD events.^16^, ^17^ Understanding the relationship between these markers of arterial change and incident HF may eventually lead to targeted therapeutic interventions.

The contributions of these subclinical vascular measures, independent of well‐established cardiovascular risk factors, on the risk of incident HF is not well understood. This can be accounted for by conflicting reports in the literature. In a study including 13 590 participants, of which 2008 developed HF, Effoe et al found that high CIMT was associated with incident HF after adjusting for demographic and traditional CVD risk factors.^12^ Additionally,^7^ in a sample of 4691 subjects with 75 cases of HF, Engström et al reported that subjects with high CIMT had an increased incidence of HF hospitalizations, even after adjusting for traditional CVD risk factors and excluding subjects with a history of myocardial infarction (MI).^18^ In contrast, in a sample of 6699 subjects with 385 cases of HF, Aladin et al found no association between CIMT and incident HF or its phenotypes after adjustment.^14^ In this study, we postulated that the noninvasive vascular measures CIMT and carotid distensibility may be related to an increased risk of incident HF independent of classic cardiovascular risk factors. We examined prospective associations between CIMT and carotid distensibility and the risk of incident HF in a cohort of older British men considering a wide range of cardiovascular risk factors.

## Methods

Data are available from the British Regional Heart Study subject to request approval (https://www.ucl.ac.uk/epidemiology‐health‐care/research/primary‐care‐and‐population‐health/research/ageing/british‐regional‐heart‐study‐brhs/brhs‐2).

The British Regional Heart Study is a prospective study of 7735 men from 24 British towns recruited and examined at baseline between 1978 and 1980, comprising a socioeconomically and geographically representative cohort. More than 99% of participants were of White European ethnicity.^19^ The cohort has been followed up by a combination of primary care record reviews, National Health Service Central Register flagging, periodic postal questionnaires, and reexaminations. A 30‐year reexamination took place in 2010 to 2012, with all 3137 surviving men invited to attend (then aged 71–92 years). Attendees completed a questionnaire, underwent physical examination, and provided a fasting blood sample.^20^ At the 30‐year follow up, a total of 2137 (68%) men completed the questionnaire, of whom 1722 (55%) men underwent a physical reexamination. All 1631 men enrolled in this study attended for a physical examination. The men were asked whether a doctor had ever told them that they had MI (heart attack, coronary thrombosis), stroke, or diabetes and to bring their medication to the examination session. Blood samples were collected after fasting for a minimum of 6 hours and were stored at −70 °C. Ethical approval has been obtained from all relevant local research ethics committees.

In this study, the components of the data used from the questionnaire include CVD risk factors, medications, and existing comorbidities. Blood pressure, body mass index (BMI), and presence of arrhythmia on ECG were used from the data collected at the physical examinations. NT‐proBNP (N‐terminal pro‐B‐type natriuretic peptide) and plasma glucose levels were used from the results obtained through blood test analysis.

### CVD Risk Factors

CVD risk factors were assessed at the 30‐year reexamination between 2010 and 2012. Information on the measurement and classification of alcohol intake, smoking status, physical activity, and social class assessed by questionnaire have already been previously described.^21^, ^22^ The use of antihypertensive medication was based on self‐reported medication history and review of codes from the British National Formulary. Anthropometric measurements of body weight and height were used to calculate the BMI as weight/(height^2^) (kg/m^2^). Blood pressure was recorded as a mean of 2 measurements done in succession in the right arm using an Omron 907 blood pressure machine (Omron, Kyoto, Japan), with the subject seated. The values used in the analysis were adjusted for observer and cuff size differences. Pulse pressure was then calculated by subtracting the mean diastolic blood pressure measurement from the mean systolic blood pressure measurement. A 12‐lead ECG was performed as part of the physical examination assessment, and ECG Minnesota codes were used in algorithms to classify arrhythmias. In this study, the atrial arrhythmias, namely, atrial fibrillation, atrial flutter, and atrial tachycardia, have been grouped together.

The study participants were asked to fast for a minimum of 6 hours before their follow‐up physical examination for fasting blood tests to be taken. NT‐proBNP was measured in plasma samples on an automated clinically validated immunoassay analyzer (e411; Roche Diagnostics, Burgess Hill, United Kingdom) using the manufacturer's calibrators and quality control reagents. The lower limit of sensitivity was 5 pg/mL. Plasma glucose was measured by a glucose oxidase method^23^ using a Falcor 600 automated analyzer. Prevalent diabetes included men with a doctor's diagnosis of diabetes and men with fasting blood glucose ≥7 mmol/L.

### Noninvasive Vascular Measures

Left and right carotid arteries were imaged by 2 experienced vascular technicians. All studies were performed using the Z.One Ultra ultrasound system (Zonare Medical Systems, Mountain View, CA) with a 5‐ to 10‐mHz linear probe. Carotid artery distension and CIMT (the distance between the leading edge of the intima and the media‐adventitia interface) were measured using the longitudinal images of the carotid artery on Carotid Analyzer Software (Medical Imaging Applications, Iowa City, IA). In the images, a 5‐ to 10 mm plaque‐free region of interest was selected, at least 1 cm from the bifurcation. Mean CIMT was calculated from intima‐media thickness measurements taken from 3 end‐diastolic images. Maximum and minimum carotid artery diameters were taken from 3 consecutive waveforms, and mean distension was calculated by subtracting baseline diameter from peak diameter. The carotid distensibility coefficient was calculated using the methods described by Dijk et al^24^ as follows: ((2×Mean distension/Baseline diameter)/Mean pulse pressure [kPa])×1000. A coefficient of variation (CV) was calculated, with respect to the inter‐ and intraobserver reproducibility for both CIMT (inter n=109, CV=7.1%; intra n=30, CV=5.1%) and carotid distension (inter n=109, CV=9.2%; intra n=30, CV=11.9%).^25^ In total, 1609 men had their CIMT measured, and 1598 men had their distensibility calculated.

### Follow‐Up

All men were followed up to June 2018 for cardiovascular morbidity and death. The mortality rate was obtained via the National Health Service Central Register. Incident HF was defined as the first occurrence of a new HF diagnosis after the 30‐year reexamination in 2010 to 2012. Incident HF was defined as a confirmed doctor's diagnosis of HF from primary care records, and verified, where possible, using clinical information from primary and secondary care records, as well as from death certificates with *International Classification of Diseases*, *Ninth Revision* (*ICD‐9*) code 428. Time to incident HF was defined as the time from the baseline reexamination in 2010 to 2012 to the incident event. For subjects who did not develop HF, the end of follow‐up was either the date of the 2018 record review or the date of death, whichever came first.

A nonfatal MI was diagnosed according to World Health Organization criteria.^26^ Evidence regarding nonfatal MI events was obtained by ongoing reports from general practitioners, by biennial reviews of the patients' practice records (including hospital and clinic correspondence) through the end of the study period and from repeated questionnaires to surviving subjects after initial examination. Fatal MI events were defined as those with death certificates with *ICD‐9* codes 410 to 414 and *ICD*, *Tenth Revision* (*ICD‐10*) codes I21 to I23.

### Statistical Analysis

In this study, we hypothesized that increased CIMT and reduced carotid distensibility may be associated with an increased risk of incident HF independent of classic cardiovascular risk factors.

The men were divided into equal quartiles based on the CIMT or distensibility distribution. We report counts with percentages for categorical variables and the correlation coefficient and *P* value for continuous variables. Cox proportional hazards modeling was used to assess the multivariate‐adjusted hazard ratios (HRs; relative risk) of incident HF by quartiles of CIMT and distensibility and per 1‐SD increase in CIMT and distensibility. Men with missing CIMT or distensibility values were excluded from their respective analyses. Men with prevalent MI were excluded from the Cox proportional hazard models for incident HF (Figure S1).

Person‐years at risk were calculated from the date of baseline until the date of incident HF. A series of models were generated, adjusting for potential confounders: age, social class (manual/nonmanual), smoking status (never/recent ex‐smoker/long‐term ex‐smoker/current), physical activity (inactive/active), alcohol (moderate/heavy drinker), BMI group (<20 kg/m^2^, 20–24.9 kg/m^2^, 25–29.9 kg/m^2^, ≥30 kg/m^2^), diabetes at baseline (yes/no), history of stroke at baseline (yes/no), prevalent MI at baseline (yes/no), taking statins at baseline (yes/no), taking antihypertensives at baseline (yes/no), arrythmia at baseline (yes/no). In multivariate analyses, blood markers were fitted as a continuous variable. HRs and 95% CIs are shown for the analyses.

All analyses were performed using SAS version 9.4 (SAS Institute, Cary, NC).

This study involves human participants who gave informed consent. Ethical approval for the British Regional Heart Study was granted by the National Research Ethics Service Committee, London Central region (reference: MREC/02/2/91). This study included only secondary data analysis.

## Results

### Baseline Characteristics

The men were divided into equal quartiles based on the CIMT or carotid distensibility distribution (quartiles 1–4). Baseline characteristics of all 1609 men who had their mean CIMT measured are given in Table 1. Compared with men with a lower mean CIMT, men in the highest mean CIMT quartile were more likely to be of a manual social class (50.37%), physically inactive (42.07%), and either a current or ex‐smoker (66.67%). They were also more likely to have cardiovascular‐related comorbidities at baseline such as stroke (11.69%), diabetes (15.94%), and cardiac arrythmia (13.35%). Men in the lowest mean CIMT quartile were more likely to have a higher mean distensibility. This inverse relationship between CIMT and carotid distensibility is emphasized in the correlation matrix given in Table 2. It shows a significant negative correlation between mean CIMT and mean distensibility (r=−0.208, *P*<0.0001). The correlation matrix shown in Table 2 also demonstrates that mean CIMT increases with age, BMI, systolic blood pressure, pulse pressure, and log–NT‐proBNP levels.

**Table 1:** Baseline Characteristics by CIMT Quartiles Among 1609* Men Without HF

Characteristic	Mean CIMT quartiles			
	1	2	3	4
	n=401	n=396	n=410	n=402
Prevalent stroke, n (%)	30 (7.5)	34 (8.6)	48 (11.7)	47 (11.7)
Prevalent MI, n (%)	63 (15.7)	37 (9.3)	62 (15.1)	52 (12.9)
Manual social class, n (%)	176 (44.1)	174 (44.1)	173 (42.3)	202 (50.4)
Never smoked, n (%)	170 (42.6)	157 (39.9)	150 (36.8)	133 (33.3)
Physically inactive, n (%)	127 (34.1)	156 (41.2)	159 (41)	159 (42.1)
Moderate/Heavy drinker, n (%)	26 (6.65)	15 (3.8)	20 (5)	13 (3.3)
Prevalent diabetes, n (%)	58 (14.8)	49 (12.7)	55 (13.7)	62 (15.9)
On antihypertensives, n (%)	241 (61.3)	229 (58.6)	249 (61.2)	230 (57.8)
On statins, n (%)	202 (50.4)	194 (49)	200 (48.8)	190 (47.3)
Atrial arrhythmias, n (%)	32 (8)	46 (11.7)	49 (12)	53 (13.4)
High carotid distensibility (top quartile), n (%)	113 (28.4)	111 (27.9)	106 (26.6)	68 (17.1)

Atrial arrhythmias include atrial fibrillation, atrial flutter or tachycardia. CIMT indicates carotid intima‐media thickness; HF, heart failure; and MI, myocardial infarction.

* Data on CIMT are missing for 22 men.

**Table 2:** Mean CIMT and Mean Carotid Distensibility Correlation Matrix

	Mean CIMT	Mean carotid distensibility
Mean CIMT		r=−0.21 *P*<0.0001
Age	r=0.19 *P*<0.0001	r=−0.30 *P*<0.0001
BMI	r=0.081 *P*=0.0012	r=−0.0034 *P*=0.89
Pulse pressure	r=0.15 *P*<0.0001	r=−0.20 *P*<0.0001
Systolic blood pressure	r=0.091 *P*=0.0003	r=−0.32 *P*<0.0001
NT‐proBNP (log)	r=0.089 *P*=0.0006	r=−0.13 *P*<0.0001

BMI indicates body mass index; CIMT, carotid intima‐media thickness; and NT‐pro BNP, N‐terminal pro‐B‐type natriuretic peptide.

Baseline characteristics of all 1598 men who had their mean carotid distensibility calculated are given in Table 3. Compared with men with a higher mean carotid distensibility, men in the lowest mean carotid distensibility quartile were more likely to be inactive (44.56%) and be either a current or ex‐smoker (67.34%). They were also more likely to have cardiovascular‐related comorbidities at baseline such as stroke (11.53%), MI (11.53%), and cardiac arrhythmias (20%). As mentioned previously, there is a negative correlation between mean CIMT and mean carotid distensibility, which is reinforced by there being more men in the lowest mean carotid distensibility quartile (39.45%) falling into the highest mean CIMT quartile, compared with the highest mean carotid distensibility quartile (17.09%). Conversely to CIMT, the correlation matrix shown in Table 2 demonstrates a negative relationship between mean carotid distensibility and age, BMI, pulse pressure, systolic blood pressure, and log–NT‐proBNP.

**Table 3:** Baseline Characteristics by Carotid Distensibility Quartiles Among 1598* Men Without HF

Characteristic	Mean carotid distensibility quartiles			
	1	2	3	4
	n=399	n=400	n=400	n=399
Prevalent stroke, n (%)	46 (11.5)	37 (9.3)	35 (8.8)	37 (9.3)
Prevalent MI, n (%)	46 (11.5)	45 (11.3)	55 (13.8)	66 (16.5)
Manual social class, n (%)	178 (44.7)	184 (46.5)	183 (45.8)	177 (44.4)
Never smoked, n (%)	130 (32.7)	157 (39.4)	149 (37.5)	169 (42.8)
Physically inactive, n (%)	168 (44.6)	150 (39.4)	147 (38.8)	132 (35.4)
Moderate/Heavy drinker, n (%)	21 (5.4)	20 (5.1)	18 (1.2)	16 (1)
Prevalent diabetes, n (%)	58 (14.8)	43 (11)	68 (17.4)	54 (14.1)
On antihypertensives, n (%)	240 (60.8)	232 (58.4)	237 (60.2)	234 (59.7)
On statins, n (%)	187 (46.9)	196 (49)	199 (49.8)	196 (49.1)
Atrial arrhythmias, n (%)	79 (20)	39 (9.8)	39 (9.8)	22 (5.6)
High CIMT (top quartile), n (%)	157 (39.5)	98 (24.5)	75 (18.8)	68 (17.1)

Atrial arrhythmias include atrial fibrillation, atrial flutter, or tachycardia. CIMT indicates carotid intima‐media thickness; HF, heart failure; and MI, myocardial infarction.

* Data on carotid distensibility are missing for 33 men.

### CIMT and Incident HF Risk

Mean follow‐up time was 6.04±1.85 years. Table 4 shows the rate of incident HF (per 1000 person‐years) by quartiles of the CIMT distribution in men without prevalent MI. The lowest mean CIMT quartile (quartile 1) was used as a reference group. The rate of incident HF increases from 6.58 per 1000 person‐years in quartile 1 to 19.7 per 1000 person‐years in quartile 4.

**Table 4:** Adjusted HRs (95% CIs) for Incident HF per CIMT Quartile

	Quartiles of mean CIMT (mm)				
	Quartile 1	Quartile 2	Quartile 3	Quartile 4	
	<0.70	0.70 to 0.78	0.78 to 0.90	0.90 to 1.79	
Total number of men	338	359	348	350	
Number of HF events	14	21	17	39	
Rate/1000 person‐years	6.58	9.27	8	19.1	

Models					Per‐unit SD* increase
Model 1, HR (95% CI)	1.0 (ref.)	1.41 (0.72–2.77)	1.06 (0.52–2.16)	2.40 (1.29–4.46)†	1.36 (1.15–1.62)†
Model 2, HR (95% CI)	1.0 (ref.)	1.30 (0.64–2.63)	0.94 (0.44–2.00)	2.20 (1.14–4.23)†	1.35 (1.12–1.63)†
Model 3, HR (95% CI)	1.0 (ref.)	1.28 (0.63–2.59)	0.95 (0.45–2.01)	1.93 (0.99–3.75)	1.30 (1.07–1.58)†

Final model variations					
Model 3a, HR (95% CI)	1.0 (ref.)	1.15 (0.57–2.34)	0.79 (0.37–1.70)	1.64 (0.84–3.21)	1.27 (1.04–1.55)†
Model 3b, HR (95% CI)	1.0 (ref.)	1.28 (0.61–2.66)	0.94 (0.43–2.05)	1.87 (0.94–3.72)	1.32 (1.08–1.59)†

Model 1: adjusted for age. Model 2: adjusted for age, social class, smoking, physical activity, alcohol intake, BMI, statin medication, prevalent diabetes, prevalent stroke, systolic blood pressure, hypertensive medication, and atrial arrhythmias. Model 3: adjusted for covariates in model 2 and carotid distensibility. Model 3a: adjusted for covariates in model 3 and time‐dependent incident MI. Model 3b: adjusted for covariates in model 3 and NT‐proBNP. BMI indicates body mass index; CIMT, carotid intima‐media thickness; HF, heart failure; HR, hazard ratio; and NT‐proBNP, N‐terminal pro‐B‐type natriuretic peptide.

* CIMT SD=0.157.

^†^ *P*<0.05.

Men in the highest CIMT quartile (quartile 4) showed a statistically significant association with incident HF risk in the age‐adjusted model (HR, 2.40 [95% CI, 1.29–4.46]; *P*=0.006) as shown in model 1 in Table 4. The significant association was maintained after adjusting for social class, smoking, physical activity, alcohol status, BMI, use of statins, prevalent diabetes, prevalent stroke, systolic blood pressure, use of antihypertensives, and atrial arrythmias (model 2: HR, 2.20 [95% CI, 1.14–4.23]; *P*=0.02). Adjustment for mean carotid distensibility (model 3) attenuated the association, resulting in an association that was not significant between mean CIMT and risk of incident HF (HR, 1.93 [95% CI, 0.99–3.75]; *P*=0.05). Subsequent adjustment of model 3 for time‐dependent incident MI (model 3a), further attenuated the association between mean CIMT and incident HF risk in men in the highest CIMT quartile (HR, 1.64 [95% CI, 0.84–3.21]; *P*=0.15). However, the continuous model, which analyzed the association per unit SD increase in CIMT, showed a statistically significant association with incident HF risk (HR, 1.27 [95% CI, 1.04–1.55]; *P*=0.02).

Adding log–NT‐proBNP to model 3 slightly weakened the association between incident HF and mean CIMT in men in the highest quartile (model 3b: HR, 1.87 [95% CI, 0.94–3.72]; *P*=0.07).

### Carotid Arterial Distensibility and Incident HF Risk

Table 5 shows the rate of incident HF (per 1000 person‐years by carotid distensibility quartiles [quartiles 1–4] in men without prevalent MI). The highest distensibility quartile (quartile 4) was used as a reference group. As mean carotid distensibility decreases, the rate of incident HF increases from 6.58 per 1000 person‐years in quartile 4 to 19.77 per 1000 person‐years in quartile 1.

**Table 5:** Adjusted HRs (95% CI) for Incident HF per Distensibility Quartile

	Carotid distensibility quartiles (×10^−3^ kPa^−1^)			
	Quartile 1	Quartile 2	Quartile 3	Quartile 4
	<9.25	9.25 to <11.75	11.75 to 14.75	>14.76
Total number of men	353	355	345	333
Number of HF events	40	20	16	14
Rate/1000 person‐years	19.77	9.21	7.3	6.58

Models					Per‐unit SD* increase
Model 1, HR (95% CI)	2.26 (1.20–4.25)†	1.22 (0.61–2.42)	1.01 (0.49–2.06)	1.0 (ref.)	0.74 (0.58–0.94)†
Model 2, HR (95% CI)	2.55 (1.24–5.24)†	1.45 (0.69–3.09)	1.13 (0.51–2.49)	1.0 (ref.)	0.71 (0.55–0.93)†
Model 3, HR (95% CI)	2.31 (1.12–4.77)†	1.39 (0.66–2.96)	1.18 (0.53–2.59)	1.0 (ref.)	0.76 (0.58–0.99)†

Final model variations					
Model 3a, HR (95% CI)	2.53 (1.23–5.22)†	1.57 (0.73–3.38)	1.32 (0.60–2.91)	1.0 (ref.)	0.73 (0.57–0.95)†
Model 3b, HR (95% CI)	3.08 (1.34–7.05)†	1.89 (0.82–4.36)	1.82 (0.75–4.41)	1.0 (ref.)	0.75 (0.57–0.98)†

Model 1: adjusted for age. Model 2: adjusted for age, social class, smoking, physical activity, alcohol intake, BMI, statin medication, prevalent diabetes, prevalent stroke, pulse pressure, hypertensive medication, and atrial arrhythmias. Model 3: adjusted for covariates in model 2 and CIMT. Model 3a: adjusted for covariates in model 3 and time‐dependent incident MI. Model 3b: adjusted for covariates in model 3 and NT‐proBNP. BMI indicates body mass index; CIMT, carotid intima‐media thickness; HF, heart failure; HR, hazard ratio; and NT‐proBNP, N‐terminal pro‐B‐type natriuretic peptide.

* Carotid distensibility SD=4.177.

^†^ *P*<0.05.

Men in the lowest carotid distensibility quartile (quartile 1) showed statistically significantly increased risk of incident HF compared with the men in the highest quartile (quartile 4) in the age‐adjusted model (HR, 2.26 [95% CI, 1.20–4.25]; *P*=0.01; Table 5, model 1). This significant association was maintained after adjusting for social class, smoking, physical activity, alcohol status, BMI, use of statins, prevalent diabetes, prevalent stroke, pulse pressure, use of antihypertensives, and atrial arrythmias (model 2: HR, 2.55 [95% CI, 1.24–5.24]; *P*=0.01). The significant association persisted after further adjustment for CIMT (model 3: HR, 2.31 [95% CI, 1.12–4.77]; *P*=0.02). Subsequent adjustment of model 3 for time‐dependent incident MI revealed a similar effect (model 3a: HR, 2.53 [95% CI, 1.23–5.22]; *P*=0.01).

Additionally, the effect was similar after adjustment of model 3 for log–NT‐proBNP (model 3b: HR, 3.08 [95% CI, 1.34–7.05]; *P*=0.008).

### CIMT, Carotid Arterial Distensibility, and Incident MI

Additional analyses were done to assess the associations between CIMT, carotid arterial distensibility, and incident MI. Following adjustment for social class, smoking, physical activity, alcohol status, BMI, use of statins, prevalent diabetes, prevalent stroke, systolic blood pressure, use of antihypertensives, and atrial arrythmias, CIMT was significantly associated with an increased risk of incident fatal and nonfatal MI per SD unit increase in CIMT (HR, 1.29 [95% CI, 1.05–1.58]). This association was not seen between carotid distensibility and incident fatal and nonfatal MI (HR, 0.9 [95% CI, 0.69–1.18]; Tables S1 and S2).

## Discussion

The objective of this study was to investigate the association between the noninvasive vascular measures CIMT and carotid distensibility and incident HF risk. In this cohort of British men aged between 71 and 92 years, we found both higher CIMT and lower carotid artery distensibility were significantly associated with higher incident HF risk, which persisted after adjustment for well‐established cardiovascular risk factors and comorbidities. The significant association between these vascular measures and incident HF were found to persist despite adjustment for each other (Table 4, model 3; Table 5, model 3). Use of either parameter may improve identification of patients at risk of developing HF.

CIMT is a measure of arterial injury.^11^, ^27^ The present study showed that increased CIMT is associated with an increased risk of incident HF, with CIMT modeled as both a continuous and categorical variable. These findings are consistent with that described by Effoe et al,^12^ who reported a significant association between CIMT and incident HF in a cohort of 13 590 American subjects. Our results were significant despite exclusion of men with prevalent MI at baseline and after adjustment for cardiovascular risk factors, comorbidities, and related medications. It has been debated in the literature that CIMT may be associated with HF through a unique mechanism different from that causing myocardial ischemia and infarction.^12^ It is reported that increased CIMT may be associated with structural vascular changes that impact arterial distensibility and therefore results in an increased systemic peripheral resistance, increased pressure afterload and eventually diastolic dysfunction leading to heart failure.^28^, ^29^ The present study found CIMT and carotid distensibility to be negatively correlated (r=−0.21, *P*<0.0001; Table 2). Additionally, adjustment of the CIMT model for carotid distensibility marginally attenuated the significance in the categorical variable analysis (quartile 4: HR, 1.93 [95% CI, 0.99–1.58]; Table 4) but not in the continuous variable analysis (SD increase: HR, 1.30 [95% CI, 1.07–1.58]; Table 4). This suggests that the association between CIMT and HF is not reliant on carotid distensibility.

Arterial distensibility is an established marker of vascular function and structure, related to arterial stiffness.^16^ In this study, we showed that reduced carotid distensibility (<9.2×10^−3^ kPa^−1^) is associated with an increased risk of HF. Our findings were significant despite exclusion of men with prevalent MI at baseline and after adjustment for cardiovascular risk factors, comorbidities including time‐dependent incident MI and related medications as well as adjustment for CIMT. Historically, arterial distensibility has been investigated in peripheral arteries (femoral and brachial) and in the aorta.^15^ Our findings contradict previously published work by Redheuil et al, who failed to find a significant relationship between aortic distensibility and incident HF in a cohort of 3675 American participants.^16^ However, Giannattasio et al reported that arterial distensibility at the level of the radial artery, carotid artery and abdominal aorta were reduced in patients with HF, and furthermore the reduction in carotid artery and aortic distensibility was directly related to the severity of HF.^30^ It has been reported that increased arterial stiffness, by way of reduced arterial distensibility, has several consequences on the cardiovascular system, which may in turn contribute to the development of HF.^31^ These consequences include increased left ventricular afterload, increased systolic blood pressure and pulse pressure, reduced diastolic blood pressure leading to reduced cardiac perfusion, and enhanced intravascular pressure resulting in premature atherosclerotic development.^16^, ^31^ An alternative measure of arterial stiffness is pulse wave velocity. Salvi et al reported a close inverse correlation between carotid–femoral pulse wave velocity and carotid distensibility, confirming a close link between these 2 measures.^32^ Numerous studies have reported a positive association between higher pulse wave velocity and HF^33^, ^34^, ^35^; however, there have been conflicting reports that pulse wave velocity is not predictive of HF in older adults.^8^ This emphasizes that the mechanisms by which arterial stiffness contributes to HF are not fully understood, necessitating the need for further research.

NT‐proBNP is a biomarker strongly associated with HF, whose synthesis predominantly occurs in ventricular myocytes in response to myocyte stretch and locally in the area surrounding an MI.^36^ Despite the widely reported relationship between NT‐proBNP and HF and the correlation shown in our study between NT‐proBNP, CIMT, and carotid distensibility (Table 2), when adjusted for in the models, NT‐proBNP weakened the association between CIMT and incident HF and strengthened the association between carotid distensibility and incident HF. This again suggests that the development of HF may be better explained by changes in arterial distensibility and also signifies that to better understand the mechanisms that could contribute to developing HF, it may be necessary in future studies to differentiate between the types of HF developed—for example, HF with preserved ejection fraction and HF with reduced ejection fraction—as the pathological pathways may differ.

In this cohort, CIMT but not carotid distensibility was associated with incident MI. The significant association between CIMT and incident MI persisted after adjustment for well‐established cardiovascular risk factors and comorbidities, which is in keeping with published literature.^37^ The lack of association between carotid distensibility and incident MI persisted despite adjustment, which suggests that carotid distensibility may be more specific to HF risk in older adults. An absence of relationship between carotid distensibility and MI has been reported previously, but those studies did not look at HF specifically.^5^, ^24^, ^38^

### Strengths and Limitations

To our knowledge, our study is the first of its kind to study the prospective relationship between CIMT and distensibility and risk of incident HF in an older British male cohort. Our study has a relatively long follow‐up, with a detailed baseline examination, which allows for adjustment for potential confounders. Our study also investigated 2 different types of vascular measures, which are markers of slightly different components of vascular aging. These measures are noninvasive, reliable, low‐cost, and easy to measure, which makes it simple to replicate in research and in clinical practice.

Excluding men with prevalent MI was key in our study because assessing the value of subclinical markers of CVD is of limited use in people with clinically apparent CVD, particularly as there is a distinct increased risk of HF in people with a history of MI.

Our definition of incident HF relies on physician‐diagnosed HF, which may underestimate the true HF incidence in the population. Despite potentially underestimating the incidence of HF, the focus of the present study was not to estimate true incidence but to assess the association between CIMT, carotid distensibility, and incident HF. The diagnostic method for HF should not affect the nature of the association between the variables. Moreover, our previously published reports exploring the associations between obesity, leptin, NT‐proBNP and incident HF,^39^, ^40^ have used the same diagnostic method for incident HF. These findings generally accord with prior data and therefore suggest potential external validity for our findings. We also did not collect data on HF subtype, and therefore we were unable to determine the type of HF more likely to be developed in association with CIMT and carotid distensibility. As previously mentioned, it may be that although the different HF subtypes may overlap with their presenting clinical features, they may have different underlying pathophysiological mechanisms.

Although our cohort was socioeconomically and geographically representative of Britain, it consisted of men and was almost entirely of White ethnic origin, which may limit the generalizability of the findings to women and people from other ethnic groups. Effoe et al conducted a similar study assessing the relationship between CIMT and incident HF in 13 590 male and female participants who self‐reported as either White or Black.^12^ They found that results were similar across race and sex groups.^12^

### Implications for Future Research

CIMT and carotid distensibility have been shown to be reliable and low‐cost measures of vascular function. Both are easily measured noninvasively in a clinical setting with ultrasound. Further longitudinal studies should report on the associations between these vascular measures and other CVD events such as incident stroke, MI, and death. It would also be of value to determine whether there is a clinically significant threshold for raised CIMT and reduced carotid distensibility and whether these are possible therapeutic targets. Additionally, it may be worth further exploring the relationship between CIMT and incident MI, specifically CIMT and carotid plaque characteristics. Future studies could also look to incorporate these measures into a prediction tool for CVD risk.

## Conclusions

Our data suggest that higher CIMT and lower carotid artery distensibility are associated with higher incident HF risk. The association between these vascular measures and incident HF were found to persist despite adjustment for established CVD risk factors and each other. Use of either parameter may improve identification of patients at risk of developing HF.

## Sources of Funding

This work was supported by the British Heart Foundation (Grant Nos. RG/19/4/34452 and PG/09/024/26857). A. A. is an Academic Clinical Fellow (Award ID: ACF‐2020‐18‐012), funded by The National Institute for Health and Care Research, UK. The views expressed are those of the authors and not necessarily those of the National Health Service, the National Institute for Health and Care Research, or the Department of Health and Social Care. The funders had no role in the design and conduct of the study; collection, management, analysis, interpretation of the data; or preparation, review, approval of, or decision to publish the manuscript.

## Disclosures

None.

## Supporting information

Tables S1–S2
Figure S1
