# Supplementary material for: Carotid Intima‐Media Thickness, Carotid Distensibility, and Incident Heart Failure in Older Men: The British Regional Heart Study
Source: J Am Heart Assoc. 2025 Mar 21;14(7):e037167. doi: 10.1161/JAHA.124.037167 (PMC12184599; doi:10.1161/JAHA.124.037167)

# Supplemental Material

**Table S1 – Adjusted hazard ratios (95% CI) for incident MI (fatal and non-fatal) per CIMT quartile**

| | Quartiles of mean CIMT (mm) | | | | |
|---|---|---|---|---|---|
| | **Q1** | **Q2** | **Q3** | **Q4** | |
| | < 0.70 | 0.70 to 0.78 | 0.78 to 0.90 | 0.90 to 1.79 | |
| Total number of men | 338 | 359 | 348 | 350 | |
| Number of MI events | 20 | 21 | 35 | 33 | |
| Rate/1000 pyrs | 7.94 | 8.38 | 14 | 13.79 | |
| **Models** | | | | | **Per unit SD increase** |
| Model 1, HR (95% CI) | 1.0 (ref.) | 1.36 (0.63, 2.93) | 1.98 (0.96, 4.05) | 2.01 (0.98, 4.13) | 1.27 (1.05, 1.54)** |
| Model 2, HR (95% CI) | 1.0 (ref.) | 1.18 (0.54, 2.57) | 1.74 (0.84, 3.62) | 1.81 (0.87, 3.78) | 1.29 (1.05, 1.58)** |

* CIMT SD = 0.157.
** P < 0.05.
Model 1: adjusted for age.
Model 2: adjusted for age, social class, smoking, physical activity, alcohol intake, BMI, statin medication, prevalent diabetes, prevalent stroke, systolic blood pressure, hypertensive medication and atrial arrhythmias.

**Table S2 – Adjusted hazard ratios (95% CI) for incident MI (fatal and non-fatal) per distensibility quartile**

| | Carotid distensibility quartiles ( x10-3 kPa-1) | | | | |
|---|---|---|---|---|---|
| | **Q1** | **Q2** | **Q3** | **Q4** | |
| | < 9.25 | 9.25 to < 11.75 | 11.75 to 14.75 | >14.76 | |
| Total number of men | 353 | 355 | 345 | 333 | |
| Number of MI events | 38 | 22 | 22 | 26 | |
| Rate/1000 pyrs | 16.28 | 8.95 | 8.67 | 10.26 | |
| **Models** | | | | | **Per unit SD increase** |
| Model 1, HR (95% CI) | 1.24 (0.65, 2.37) | 0.88 (0.44, 1.74) | 0.87 (0.43, 1.74) | 1.0 (ref.) | 0.89 (0.69, 1.15) |
| Model 2, HR (95% CI) | 1.08 (0.54, 2.17) | 0.88 (0.44, 1.79) | 0.80 (0.39, 1.65) | 1.0 (ref.) | 0.90 (0.69, 1.18) |

* Carotid distensibility SD = 4.177.
**P < 0.05.
Model 1: adjusted for age.
Model 2: adjusted for age, social class, smoking, physical activity, alcohol intake, BMI, statin medication, prevalent diabetes, prevalent stroke, pulse pressure, hypertensive medication and atrial arrhythmias.

**Figure S1. Flowchart demonstrating study participant selection.**

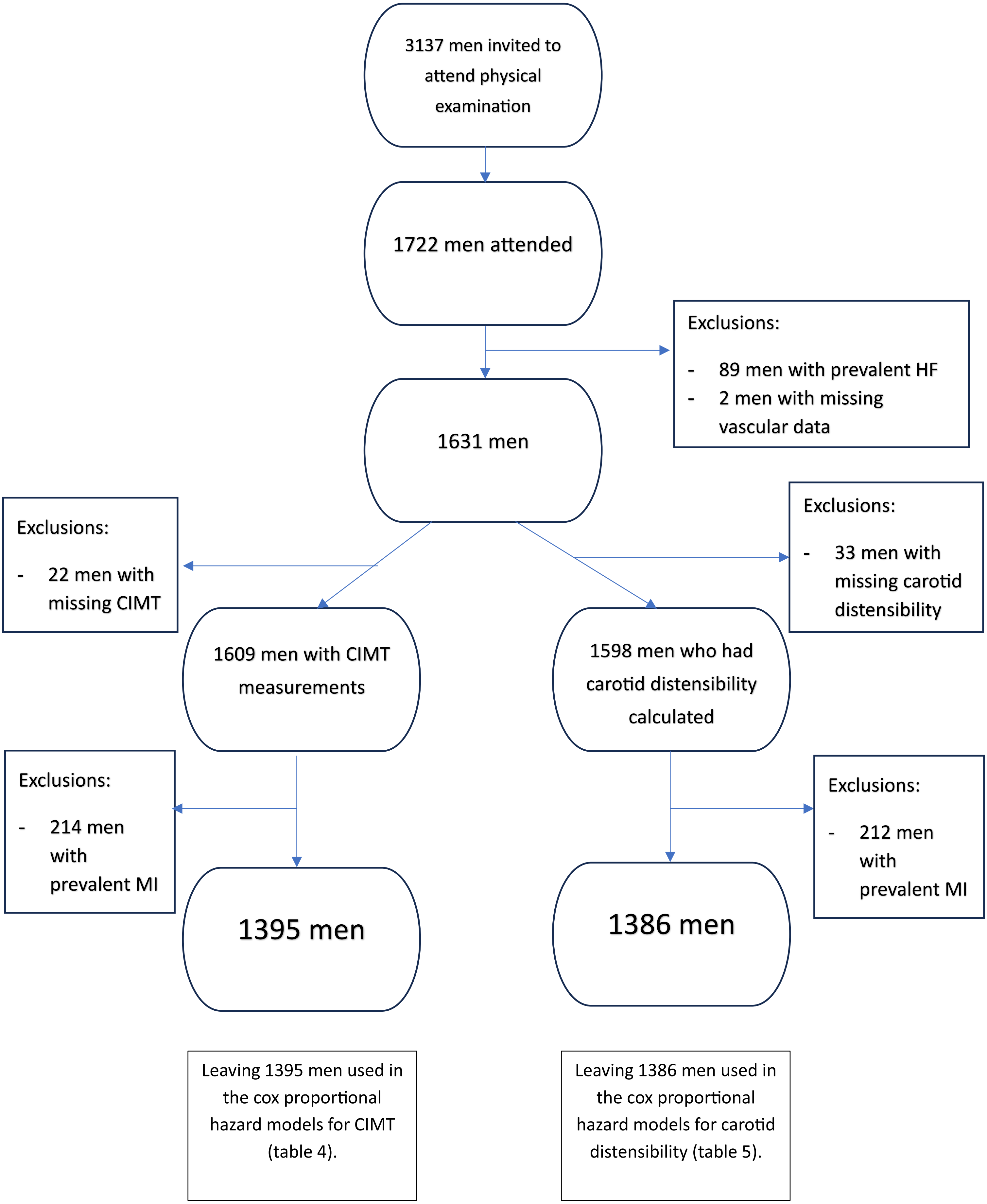

Supplement: Supplementary file 1 — Tables S1–S2 Figure S1 [file JAH3-14-e037167-s001.pdf]
